# Sulbactam-durlobactam plus ceftriaxone dosing and novel treatment regimens for *Mycobacterium abscessus* lung disease

**DOI:** 10.1128/aac.01437-25

**Published:** 2026-04-09

**Authors:** Sanjay Singh, Avneesh Shrivastava, Gunavanthi D. Boorgula, Mary C. Long, Brian Robbins, Pamela J. McShane, Tawanda Gumbo, Shashikant Srivastava

**Affiliations:** 1 Division of Infectious Diseases, Department of Medicine, School of Medicine, University of Texas at Tyler 12347, Tyler, Texas, USA; 2 Advanta Genetics 734520 , Tyler, Texas, USA; 3 Section of Pulmonary and Critical Care, Department of Medicine, School of Medicine, University of Texas at Tyler 12347, Tyler, Texas, USA; 4 IMPI Group of Companies, Mt. Hampden, Zimbabwe; 5 Phase Advance, Dallas, Texas, USA; 6 Department of Cellular and Molecular Biology, University of Texas Health Science Centre at Tyler 12341, Tyler, Texas, USA; Queen Mary University of London, London, United Kingdom

**Keywords:** target redundancy, “double-double” β-lactam-β-lactamase inhibitors, probability of target attainment, *γ* slopes

## Abstract

The guideline-based therapy achieves sputum culture conversion rates in 20%–34% of patients with *Mycobacterium abscessus* (MAB) lung disease (LD). Double-β-lactam combinations have been proposed to improve cure based on time-kill curves. We tested MICs followed by hollow fiber system model of MAB (HFS-MAB) exposure-effect studies with sulbactam-durlobactam administered every 8 h (q8h), q12h, and q24h, to identify target exposures. Next, the sulbactam-durlobactam target exposure plus ceftriaxone was administered in the HFS-MAB inoculated with three different MAB isolates, as was the sulbactam-durlobactam-ceftriaxone combination with epetraborole and omadacycline (SDCEO). *γ*-slopes (kill-speed) were calculated for all regimens. The minimal sulbactam-durlobactam clinical doses that achieved target exposure were identified using Monte Carlo experiments. Ceftriaxone reduced sulbactam-durlobactam MICs by eight-tube dilutions. In the HFS-MAB, sulbactam-durlobactam microbial kill and antimicrobial resistance were linked to % time concentration persists above MIC (%T_MIC_), with target exposure of 50%T_MIC_. Sulbactam-durlobactam killed 3.85 log_10_ CFU/mL below day 0 burden (*B_0_*) with regrowth. Sulbactam-durlobactam plus ceftriaxone killed without regrowth and demonstrated Bliss additivity. The kill slope of bacterial population in >95% of virtual subjects was 2.28 (0.97–4.80) log_10_ CFU/mL/day for sulbactam-durlobactam-ceftriaxone and 2.91 (1.65–4.93) log_10_ CFU/mL/day for SDCEO. The optimal sulbactam-durlobactam dose co-administered with ceftriaxone was 2G q8h for creatinine clearance >90 mL/min (or 4G q12h for outpatients), 2G q12h for 60–90 mL/min, 1G q12h for ≥30 to <60 mL/min, and 1G q24h for <30 mL/min. Sulbactam-durlobactam-ceftriaxone achieved the highest microbial kill encountered so far in the HFS-MAB. Sulbactam-durlobactam-ceftriaxone should be tested as the backbone for novel treatment shortening regimens.

## INTRODUCTION

*Mycobacterium abscessus* (MAB) lung disease treated with guideline-based therapy achieves sputum culture conversion in only 23%–34% of patients (1). The β-lactam antibiotics, cefoxitin and imipenem, are the backbone of guideline-based therapy (2). The poor success of guideline-based therapy is because the MAB cell wall is relatively impermeable to antibiotics, and antimicrobial resistance emergence is almost universal (3, 4). The MAB cell wall is composed of an outer mycolic acid layer, a middle arabinogalactan layer, and the inner peptidoglycan layer. Cell wall biogenesis in MAB is a four-step process; the second step is peptidoglycan septum synthesis (5, 6). β-Lactams inhibit the peptidoglycan synthesis by inactivating penicillin-binding proteins (PBPs), such as D,D-transpeptidases, L,D-transpeptidases 1 to 5 (Ldt_Mab1_ to Ldt_Mab5_), the bifunctional transglycosylase, the D,D-transpeptidase PonA1, and D,D-carboxypeptidases (D,D-c) (5–8). Therefore, “target redundancy,” that is, simultaneous targeting of all the PBPs, has been proposed as a therapeutic strategy (9).

MABs harbor the β-lactamase, Bla_Mab_, and therefore, β-lactamase inhibitors (BLIs), such as sulbactam (a β-lactam) and durlobactam (a diazabicyclooctane) represent an attractive strategy to combat antimicrobial resistance (10, 11). The BLIs and β-lactams have varying affinities for Bla_Mab_ and PBPs (7, 10–14). As reported elsewhere, ceftriaxone and cefotaxime inactivate PonA2, PonA1, and D,D-c PbpA at low concentrations, while ceftazidime and cefoxitin inactivate the same at intermediate concentrations, albeit requiring higher concentrations of the BLI, sulbactam (14). Recent studies found that another BLI, durlobactam, inactivates Bla_Mab_ and all Ldt_Mab_ (except Ldt_Mab3_) more potently (15). Therefore, a target redundancy strategy has been proposed for durlobactam, sulbactam, imipenem, cefuroxime, ceftaroline, and ceftazidime (7, 9, 16–19).

Here, we tested ceftriaxone plus sulbactam-durlobactam against multiple clinical isolates of MAB. We also tested experimental combinations with epetraborole, omadacycline, and minocycline, with elsewhere reported antimicrobial activity of these drugs against MAB (20–22). Epetraborole and omadacycline were chosen because they achieve microbial kill below day 0 bacterial burden (*B_0_*) that is 15-fold and 7.8-fold better than guideline-based therapy, while minocycline is part of guideline-based therapy (20–24). Since the shape of the concentration-time curve is crucial to β-lactam efficacy, and an oscillating stressor (concentration-time profiles with repetitive dosing) more easily generates antimicrobial resistance, we conducted a series of studies in which we mimicked intrapulmonary pharmacokinetics (PKs) of drugs in the preclinical hollow fiber system model of MAB lung disease (HFS-MAB) (25–38). The HFS-MAB offers repetitive sampling, thus enabling tracking of MAB burdens (CFU/mL) treated with different drug exposures over time. The HFS-MAB fits the “FDA Roadmap to Reducing Animal Testing in Preclinical Safety Studies” approach and the NIH initiative to phase out animal models for therapeutics for human diseases, as do the tandem *in silico* translations (39, 40).

## RESULTS

### MICs

**Fig 1:**
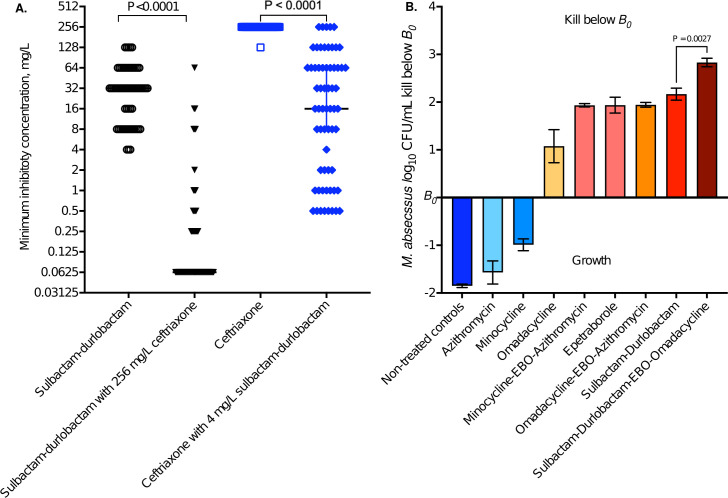
Minimal inhibitory concentrations and time-kill with different drugs and combinations. All assays were performed in triplicate. (**A**) MICs were performed for 63 MAB isolates, under four conditions: sulbactam-durlobactam, sulbactam-durlobactam with 256 mg/L ceftriaxone (trio), ceftriaxone, and ceftriaxone plus 4 mg/L sulbactam-durlobactam (trio). Shown are the reductions in MIC for trios. (**B**). Time-kill curves with static concentrations. EBO is epetraborole.

The MICs of sulbactam-durlobactam alone, ceftriaxone alone, and sulbactam-durlobactam plus ceftriaxone for each MAB clinical isolate are listed in Table S1 and summarized in Fig. 1A. The sulbactam-durlobactam cumulative MIC for 50% of clinical isolates (MIC_50_) was 32 mg/L, and the MIC_90_ was 64 mg/L. When co-incubated with a fixed ceftriaxone concentration of 256 mg/L, the sulbactam-durlobactam MICs were <0.125 mg/L (for calculation purposes categorized as 0.06 mg/L), leading to MIC_50_ of 0.06 mg/L and an MIC_90_ of 8 mg/L. Thus, ceftriaxone reduced sulbactam-durlobactam MICs by a median of eight-tube dilutions (*P* < 0.001).

The ceftriaxone MICs are shown in Fig. 1A. The ceftriaxone MIC_50_ and MIC_90_ were >128 mg/L (graphed as 256 mg/L). However, in the presence of a fixed sulbactam-durlobactam concentration of 4 mg/L, the ceftriaxone MIC_50_ was 1 mg/L, and MIC_90_ was 64 mg/L. The ceftriaxone MICs in the presence of sulbactam-durlobactam correlated with sulbactam-durlobactam MICs (*P* < 0.001) with a Pearson r of 0.42 (95% confidence interval [CI] 0.18–0.61). This means that the ceftriaxone MICs were partially driven by the sulbactam-durlobactam effective concentration. In addition, even though the sulbactam-durlobactam MIC_50_ of 32 mg/L was higher than 4 mg/L used in the assay, sulbactam-durlobactam still reduced ceftriaxone MICs. In short, sulbactam-durlobactam reduced ceftriaxone MICs by four-tube dilutions (*P* < 0.001).

### Static time-kill studies

Figure 1B shows the effects of different drugs, including sulbactam-durlobactam, with 72 h of co-incubation with MAB. Azithromycin and minocycline did not kill below B_0_. Sulbactam-durlobactam duo killed 2.17 ± 0.13 log_10_ CFU/mL below *B_0_,* better than the macrolide and tetracycline-containing combinations. Sulbactam-durlobactam combination with epetraborole and omadacycline killed 2.83 ± 0.09 log_10_ CFU/mL below *B_0_*, better than sulbactam-durlobactam duo.

### Sulbactam-durlobactam exposure-effect and dose scheduling study in the HFS-MAB

The observed drug concentration-time profiles and PKs for sulbactam are shown in Fig. S1A through D, while for durlobactam, are shown in Fig. S2A through D. The PK modeled data for sulbactam and durlobactam (Table S2) were used to calculate AUC_0-24_/MIC ratios, peak concentration to MIC ratio, and time concentration persists above MIC (%T_MIC_); the MAB ATCC#19977 inoculated in HFS-MAB had an MIC of 4 mg/L for sulbactam-durlobactam (1:1 combination).

**Fig 2:**
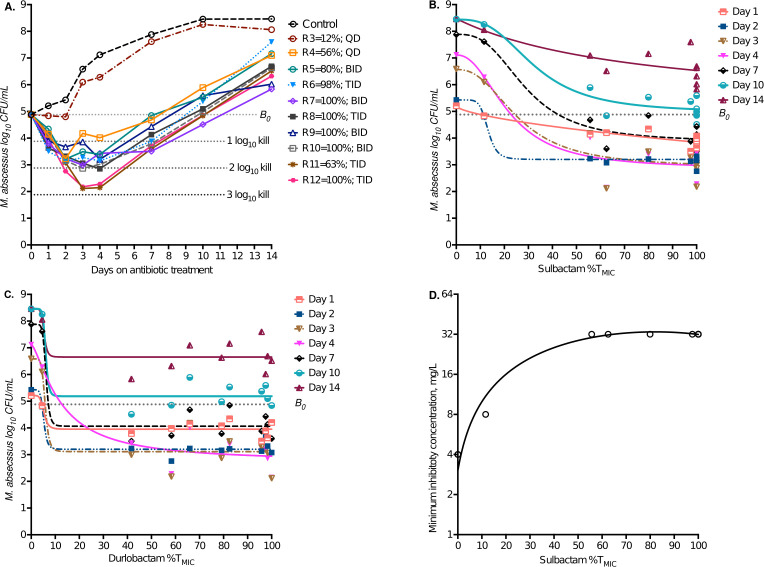
Sulbactam-durlobactam pharmacokinetics/pharmacodynamics in the HFS-MAB. (**A**) Changes in bacterial burden between sampling days on different treatment regimens, shown with the percentage of dosing interval sulbactam concentrations stayed above MIC. All treatment regimens demonstrated a biphasic effect. (**B**) Inhibitory sigmoid E_max_ curves based on sulbactam exposure, for the different sampling days. (**C**) Inhibitory sigmoid E_max_ curves based on durlobactam exposure, for the different sampling day, show that steep portions of the curves were between 0 and 10% exposures. (**D**) Quadratic function for MICs versus sulbactam exposure. (Sul/Dur, sulbactam/durlobactam)

The day-to-day sampling of HFS-MAB units revealed changes in bacterial burden shown in Fig. 2A. The bacterial burden on day 0 (inoculum or *B_0_*) was 4.88 log_10_ CFU/mL. In non-treated HFS-MAB units, bacterial burden grew to 8.46 log_10_ CFU/mL on day 14. The highest microbial kill below *B_0_* occurred on day 3. After day 4, the bacterial burden began to increase. Since sulbactam degrades at 37°C when supplemented in agar, we could not quantify the drug-resistant CFU/mL. However, MICs of samples from each HFS-MAB unit on day 14 demonstrated that while the MIC did not change in non-treated controls (4 mg/L), it increased by one-tube dilution (8 mg/L) in the lowest exposure and three-tube dilutions (32 mg/L) in the rest of the sulbactam-durlobactam-treated HFS-MAB units.

The PK/PD driver for microbial kill for sulbactam was %T_MIC_, as shown in Table S3. For durlobactam, microbial kill %T_MIC_ was also the driver, except on day 2 when AUC_0-24_/MIC was the PK/PD driver. The relationship between sulbactam %T_MIC_ and total bacterial burden is shown in Fig. 2B, while that for durlobactam is shown in Fig. 2C. The inhibitory sigmoid E_max_ parameter estimates are shown in Table 1, as are the target exposures (EC_80_) for each day. The mean sulbactam target exposure was %T_MIC_ of 49.95 ± 8.43% or ~50%, while that for durlobactam was 10.5±3.8%. When examined using microbial kill below *B_0_*, the maximal kill from the model was 3.85 ± 1.3 log_10_ CFU/mL, on day 4. As regards to antimicrobial resistance, the relationship between sulbactam %T_MIC_ and MIC on day 14 is shown in Fig. 2D (r^2^ > 0.99) was associated with an Akaike information criteria score of 17.16, as compared with 51.35 for AUC_0-24_/MIC, and 51.04 for C_max_/MIC. The lower the Akaike information criteria score, better is the model fit. This means %T_MIC_ was also a PK/PD driver for sulbactam-durlobactam antimicrobial resistance. Results were similar for durlobactam versus MIC (r^2^ = 0.98).

**TABLE 1:** Inhibitory sigmoid E_max_ model parameter estimates for sulbactam and durlobactam on different sampling days^*a*^

	E_con_ log_10_ CFU/mL	E_max_ log_10_ CFU/mL	H	EC_50_ %T_MIC_	Target exposure (EC_80_) %T_MIC_	r^2^
Sulbactam						
Day 1	5.02	1.31	2.20	45.86	86.24	0.82
Day 2	5.43	2.23	7.19	13.18	15.98	0.93
Day 3	6.57	3.67	2.43	26.16	46.24	0.83
Day 4	7.11	4.27	2.25	21.90	40.52	0.90
Day 7	7.89	4.04	2.82	29.04	47.48	0.93
Day 10	8.44	3.49	2.90	32.70	52.79	0.93
Day 14	8.47	3.45	2.61	41.19	70.12	0.65
Durlobactam						
Day 1	5.21	1.26	6.04	5.23	6.58	0.76
Day 2	5.43	2.23	7.17	5.18	6.28	0.93
Day 3	6.59	3.47	7.32	5.82	7.04	0.82
Day 4	7.12	4.40	1.41	12.44	33.16	0.90
Day 7	7.88	3.82	8.56	6.15	7.23	0.92
Day 10	8.46	3.27	9.82	6.00	6.91	0.91
Day 14	8.46	1.81	8.05	5.31	6.31	0.63

^*a*^ E_con_, effect in non-treated control; E_max_, maximal effect; EC_50_, exposure mediating 50% of E_max_.

### Combination therapy studies using multiple clinical isolates in the HFS-MAB

The three MAB clinical isolates had MICs shown in Table 2. The drug concentration-time profiles measured in the combination therapy HFS-MAB studies are shown in Fig. S3. The exposures achieved for each drug are shown in Table S4.

**TABLE 2:** Drug minimum inhibitory concentrations (MIC in mg/L) change with treatment in HFS-MAB

	Day 0	Day 14			
Regimen (R)	Inoculum	Sulbactam-durlobactam (R1)	Ceftriaxone (R2)	Sulbactam-durlobactam-Ceftriaxone (R3)	Non-treated (R4)
Isolate #1 (MAB_2)					
Sulbactam-durlobactam	8	**16^*b*^**	8	**16**	8
Ceftriaxone	**>256**	**>256**	**>256**	**>256**	>256
Sulbactam-durlobactam + 256 mg/L ceftriaxone	**≤0.125**	**≤0.25**	**≤0.25**	**≤0.25**	≤0.25
Ceftriaxone + 4 mg/L sulbactam-durlobactam	0.5	**16**	8	**32**	8
Isolate #2 (MAB_12)^*a*^					
Sulbactam-durlobactam	16	16	16	**128**	16
Ceftriaxone	>256	>256	>256	>256	>256
Sulbactam-durlobactam + 256 mg/L ceftriaxone	<0.125	**0.5**	≤0.25	**32**	≤0.25
Ceftriaxone + 4 mg/L sulbactam-durlobactam	8	**64**	16	**256**	16

^*a*^ The same isolate was used in the two-drug and multi-drug combination HFS-MAB studies.

^*b*^ Bold, change in MIC indicating accquired drug resistance.

**Fig 3:**
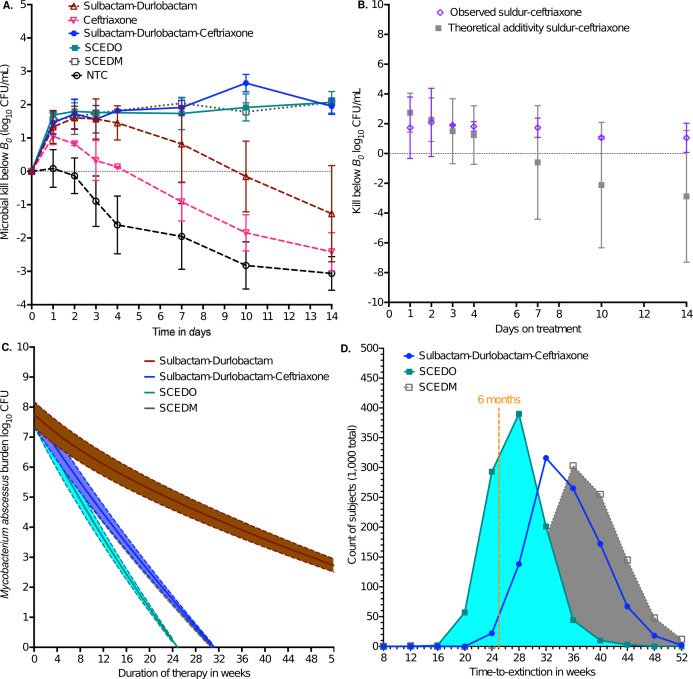
Combination therapy pharmacodynamics and bacterial population extinction. SDCEM is sulbactam-durlobactam-ceftriaxone plus epetraborole plus minocycline; SDCEO is sulbactam-durlobactam-ceftriaxone plus epetraborole plus omadacycline; NTCs are non-treated controls. (**A**) Kill below day 0 bacterial burden (*B_0_*) for the different regimens. Symbols show mean value, and error bars standard deviation. (**B**) Error bars are 95% confidence intervals; symbols are mean values. (**C**) Modeling of bacterial burden decline till extinction, showing the median and 95% credible intervals. (**D**) Time to extinction is shown for those regimens that achieved that at or before 52 weeks.

Given that each MAB clinical isolate achieved different *B_0_*, and non-treated HFS-MAB units achieved different bacterial burdens (*B_x,t_*) from each other on each sampling day (*t*) due to differences in growth rates, we modeled the therapy effect as kill below *B_0_*, with results shown in Fig. 3A. Kill below *B_0_* is a positive number, and growth above *B_0_* is a negative number. Fig. 3A shows that while ceftriaxone monotherapy had minimal effect. Sulbactam-durlobactam duo killed MAB clinical isolates better than ceftriaxone, but there was rebound growth. In addition, Fig. 3A also shows that the sulbactam-durlobactam-ceftriaxone combination trio was more effective and killed about 2 log_10_ below *B_0_* and then held the bacterial burden constant without rebound growth. Furthermore, Fig. 3A shows that all the sulbactam-durlobactam-ceftriaxone combinations with epetraborole or tetracyclines (omadacycline [SDCEO] versus minocycline [SDCEM]) killed more than sulbactam-durlobactam-ceftriaxone trio, though the effect was not statistically different for SDCEM. In Fig. 3B, we compared the observed sulbactam-durlobactam-ceftriaxone trio kill below *B_0_* to the sum of ceftriaxone monotherapy plus sulbactam-durlobactam duo (theoretical Bliss-type additivity). The observed trio effect fell within the 95% confidence interval of the theoretical additivity. This means that the effects of sulbactam-durlobactam and ceftriaxone were additive and not antagonistic. As regards resistance, the MICs on day 14, at the end of the experiments, are shown in Supplementary Results and Table 2.

### *γ*, time-to-extinction, and time to cure

The results of all MAB isolates (including from the reference laboratory strain ATCC#19977) were combined and co-modeled for *γ* for each of the six treatment regimens used in the HFS-MAB studies, with results shown in Fig. 3C, and Table 3. Figure 3C shows that the model sampled from a *B_0_* between 7.0 and 8.0 CFU. *γ* is the speed of kill and ranked by this parameter the regimen with the fastest kill was SDCEO, followed by SDCEM. The predicted time-to-extinction is shown in Fig. 3D. The % of virtual subjects with MAB population extinction at 52 weeks (1 year) was 0.9% for ceftriaxone monotherapy, 38.4% for sulbactam-durlobactam, 99.9% for sulbactam-durlobactam-ceftriaxone, 98.9% for SDCEM, and >99.9% for SDCEO. For sulbactam-durlobactam-ceftriaxone, the mean time-to-extinction was 34.43 (95% CI: 34.1–34.75) weeks, SDCEM 37.8 (95% CI: 37.39–38.21) weeks, and for SDCEO 27.15 (95% CI: 26.91–27.4) weeks. As regards sulbactam-durlobactam or ceftriaxone, the mean time to extinction was >52 weeks. When analysis was performed for time to extinction for >95% of the virtual subjects (i.e., time-to-cure), the results were as shown in Table 3. SDCEO was predicted to achieve that in 33.75 weeks, while sulbactam-durlobactam-ceftriaxone was predicted to achieve the cure at 43.75 weeks.

**TABLE 3:** Ordinary differential equation parameter estimates and 95% credible intervals^*a*^

	*B_0_* log_10_ CFU/cartridge	*r* log_10_ CFU/ml/day	K_max_ log_10_ CFU/cartridge	*γ* log_10_ CFU/ml/day	Time to extinction in 95% of subjects in weeks
Sulbactam-durlobactam	6.14 (5.22–6.32)	0.18 (0.05–0.32)	10.15 (8.94–26.5)	1.36 (0.74–3.06)	>52
Ceftriaxone	5.56 (5.13–5.96)	0.17 (0.11–0.25)	9.59 (8.93–10.72)	0.94 (0–2.81)	>52
Sulbactam-durlobactam + ceftriaxone	6.62 (6.01–7.21)	0.16 (0.07–0.29)	10.37 (9.48–13.11)	2.28 (0.97–4.80)	43.75 (31.12–57.92)
Sulbactam-durlobactam + ceftriaxone + epetraborole + omadacycline (SDCEO)	8.03 (7.74–8.31)	0.12 (0.07–0.18)	11.35 (10.74–12.69)	2.91 (1.65–4.93)	33.75 (23.55–37.5)
Sulbactam-durlobactam + ceftriaxone-epetraborole + minocycline (SDCEM)	8.03 (7.74–8.31)	0.12 (0.07–0.18)	11.35 (10.74–12.69)	2.30 (0.80–4.83)	37.8 (95% CI: 37.39–38.21)
All isolates	6.87 (6.3-7.41)	0.15 (0.06-0.27)	10.57 (9.69-13.33**)**	–^*b*^	–

^*a*^ *B*_*0*_, the pretreatment bacterial burden; k_max_ , the carrying capacity, which is the maximum possible bacterial population carried by hollow fiber system cartridge; 𝜸, the kill rate or slope from ordinary differential equation.

^*b*^ –, not applicable.

### Probability of target attainment (PTA) for clinical dose selection

Monte Carlo experiments (MCEs) were performed in a total of 480,000 virtual subjects for sulbactam and 480,000 for durlobactam, across a range of creatinine clearances. The PK output in the virtual subjects was compared with that used in the domain of input of the MCE in Table S5. The MCE thus recapitulated PKs observed in the clinic well.

**Fig 4:**
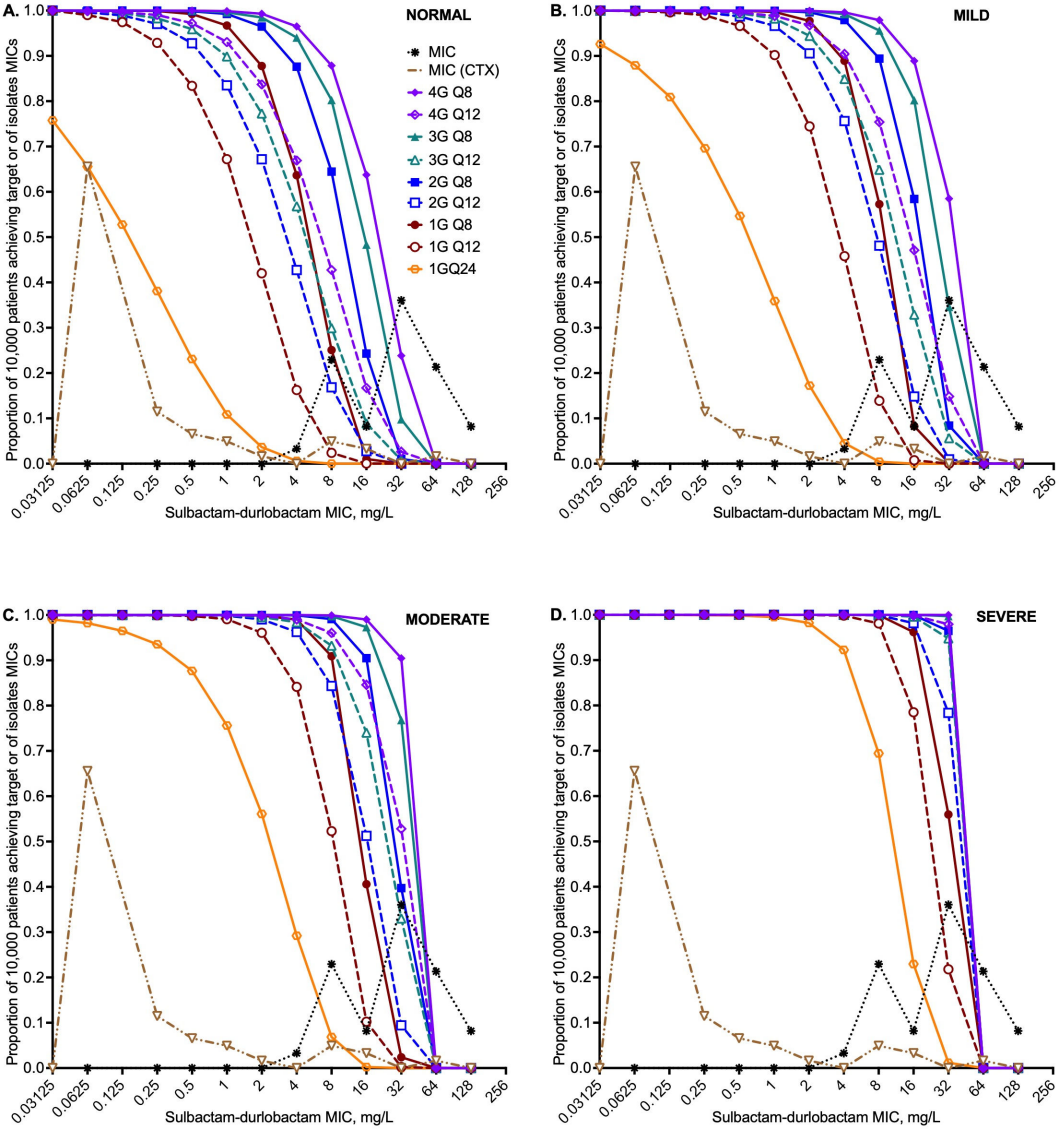
Probability of target attainment for sulbactam. MIC (CTX) refers to sulbactam-durlobactam MIC with 256 mg/L of ceftriaxone. Every 8 h dosing (q8h) is shown with solid symbols and lines, every 12 h (q12h) open symbols and hatched lines, and the 1G q24h dose is the hexagon. (**A**) Probability of target attainment (PTA) in 10,000 virtual subjects with creatinine clearance of >90 mL/min. (**B**) PTA in 10,000 virtual subjects with creatinine clearance of 60–90 mL/min, which is mild renal dysfunction. (**C**) PTA in 10,000 virtual subjects with creatinine clearance of ≥30 to <60 mL/min. (**D**) PTA in 10,000 virtual subjects with creatinine clearance of <30 mL/min.

The sulbactam probability of target attainment (PTA) is the proportion of virtual patients achieving the target exposure of %T_MIC_ of 50% in the lung at each MIC for the different creatinine clearance categories as shown in Fig. 4. We observed that the PTA improved as renal dysfunction worsened, as shown from considering the PTA for the lowest dose of 1G q24h at the MIC of 2 mg/L, which was 1% with normal renal function (Fig. 4A), 9% with mild (Fig. 4B), 40% with moderate (Fig. 4C), and 96% with severe renal dysfunction (Fig. 4D). The two MIC distributions for sulbactam-durlobactam without and with a fixed ceftriaxone concentration of 256 mg/L are also shown in Fig. 4. The q8h dosing schedule achieved sulbactam PTA ≥90% above the MIC_90_ of MIC distribution sulbactam-durlobactam with ceftriaxone versus at the MIC_10_ of MIC without ceftriaxone for normal renal function. This means that renal function, dosing schedule, and use of ceftriaxone are important drivers for achievement of >90% PTA for sulbactam. The PTAs for durlobactam are shown in Fig. S4A through D.

**Fig 5:**
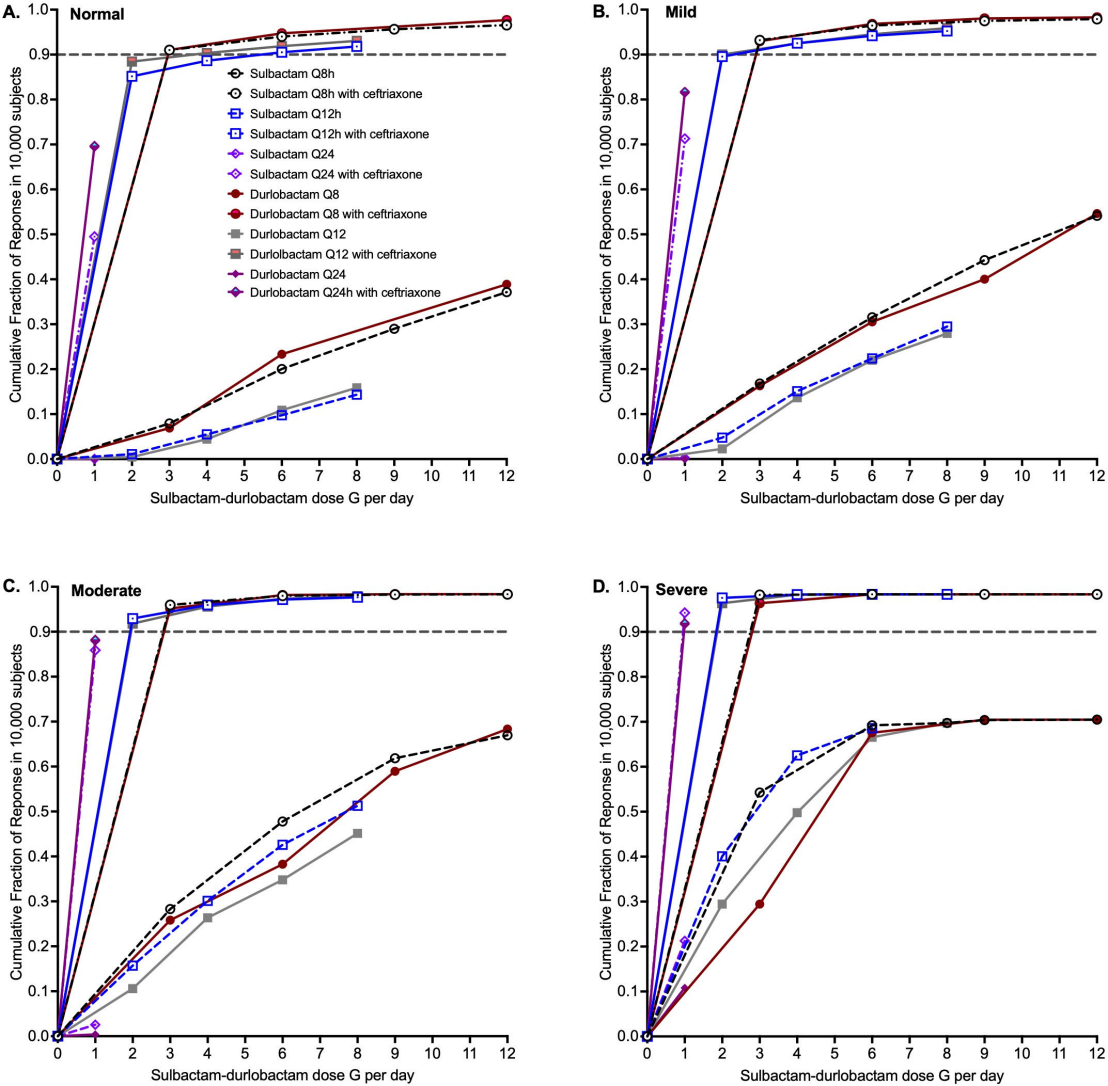
Cumulative fraction of response for both sulbactam and durlobactam. Cumulative fraction of response (CFR) in patients for both sulbactam target exposure (%T_MIC_ = 50%) and durlobactam exposure target (%T_MIC_ = 10.5%). Figure legends are shown in panel D. Open symbols and hatched lines are for sulbactam, while closed symbols and solid lines are for durlobactam. Circles are for q8h dosing, squares q12h, and diamonds q24h. Pairs with and without ceftriaxone are color-coordinated. The proportion of 90% of virtual patients achieving target attainment across all MICs is shown by the gray hatched line. (**A**) CFR in 90,000 virtual patients with creatinine clearance >90 mL/min. (**B**) CFR in 90,000 virtual patients with creatinine clearance 60–90 mL/min. (**C**) CFR in 90,000 virtual patients with creatinine clearance ≥30 to <60 mL/min. (**D**) CFR in 90,000 virtual patients with creatinine clearance <30 mL/min.

The cumulative fraction of response (CFR), which is the proportion of virtual patients that achieve target exposures when all MICs are summated, is shown by renal function in Fig. 5A through D. The lowest dose that achieves CFR in >90% of virtual patients is the best dose for the clinic (i.e., optimal dose). Since durlobactam CFR was achieved at much lower doses compared with sulbactam, the sulbactam CFR values were used to define the optimal sulbactam-durlobactam dose. Figure 5 shows that under all the different degrees of renal function, the co-administration of sulbactam-durlobactam with ceftriaxone achieved the target in a larger proportion of virtual patients compared with when there was no co-administration of ceftriaxone. Indeed, all the doses based on sulbactam-durlobactam without ceftriaxone failed to achieve the target in 90% of virtual patients even up to 4G q8h (12 g per day). Figure 5 also shows that the lowest durlobactam dose of 1 g q24h achieved a CFR >90% for all, regardless of renal function. Thus, the lowest doses in which both sulbactam and durlobactam achieved CFR ≥90% were chosen based on sulbactam CFRs. In patients with normal renal function, 2G q8h (or 4G q12h) were the optimal doses (Fig. 5A), no sulbactam q12h or q24h doses achieved a CFR >90%. In Fig. 5B, at a creatinine clearance of 60–90 mL/min, the sulbactam CFR was >90% at the lowest doses of 1G q8h or 2G q12h. At a creatinine clearance of ≥30 to <60 mL/min (Fig. 5C), sulbactam 1 g q12h was the optimal dose. With creatinine clearance less than 30 mL/min (Fig. 5D), 1G q24h was optimal. This dosing (in inpatient and outpatient settings) is summarized in Table S6 for clinical use.

## DISCUSSION

Thirty-five percent of patients with MAB lung disease present with cavitary disease, and the presence of cavities with diameter greater than 2 cm is associated with up to 2.5 times higher risk of death on treatment (41, 42). This is because the pretreatment bacterial burden (*B_0_*) in lung cavities is high, between 7.0 and 8.0 log_10_ CFU/lung (43, 44). *B_0_* has been shown to be a major determinant of therapy outcomes, consistent with the inoculum effect in PK/PD studies (43, 44). Therefore, drugs for the target redundancy strategy must be chosen based on how extensively they kill MAB below *B_0_* (24, 27, 29, 45). In order to improve on guideline-based therapy, ideally, drugs chosen must kill below *B_0_* more extensively than the standard of care. In comparing sulbactam-durlobactam kill below *B_0_* in the HFS-MAB to other β-lactams, such as cefoxitin, imipenem/relebactam, and ceftaroline, for combination in a double β-lactam regimen, sulbactam-durlobactam ranks highest, and even outranks guideline-based therapy (24, 27, 29, 45). In addition, ceftriaxone has an 8-h half-life, while sulbactam-durlobactam has a half-life of 2.5 h, which we recapitulated in the HFS-MAB here, which could lead to once or twice a day dosing, more practical than imipenem (half-life 1h) and cefoxitin (half-life <1 h) that are administered four to six times a day (27, 29, 45). These considerations, together with mechanistic data on affinity to the different PBPs, inform us on which drugs among β-lactams to combine and test in the clinic.

In the present study, ceftriaxone and sulbactam-durlobactam reduced each other’s MICs by multiple tube dilutions, essentially providing an opportunity to achieve the target % of time above MIC at the site of infection with infrequent dosing schedule. However, these findings also highlight the need to develop MIC assays for the “double β-lactam” combinations and for identification of PK/PD breakpoint values and the MIC distribution of isolates from patients’ sputa. From a clinical microbiology laboratory perspective, this can be accomplished in MIC assays by adding a fixed concentration of one drug (either sulbactam-durlobactam or ceftriaxone) throughout the wells and then varying the concentration of the companion drug through a range of concentration series. However, this will require further work and additional manpower and resources, resulting in added cost of care.

Previous static concentration-based time-kill curves studies have shown that sulbactam-durlobactam alone and in combination with ceftaroline, cefuroxime, cefoxitin, and imipenem, kill MAB more than guideline-based therapy (46). This effect was confirmed by time-kill curves in the present study, as shown in Fig. 1B. However, time-kill studies have no straightforward translation to clinical dosing schedules and cannot be used to identify % of time above MIC target exposures, because the exposures are either 0 or 100% in time-kill assays. To this end, the dynamic HFS-MAB mimics human intrapulmonary PKs in order to identify (i) the target exposure and hence optimal clinical dose and (ii) optimal dose schedule that drives microbial kill and antimicrobial resistance emergence. The HFS-MAB allowed us to identify the dosing schedule as linked to %T_MIC_, as well as two different target exposures: 50% for sulbactam and 10.5% for durlobactam. Moreover, we demonstrated that ceftriaxone was additive to sulbactam-durlobactam with regard to microbial kill on all sampling days in the HFS-MAB, while preventing rebound growth.

Another advantage of the HFS-MAB is that there is a straightforward pathway to translating results to clinical doses, based on standard PK/PD approaches and Monte Carlo experiments. Indeed, this is one of the primary uses of PK/PD science and is the one recommended by regulatory authorities for clinical dose selection (47, 48). As an example, Monte Carlo experiment-derived dose finding for omadacycline and imipenem using HFS-MAB output was shown to be accurate based on real-world clinical data from patients treated for MAB lung disease (21). One of our main goals was taking the concept of “target redundancy” from the test tube to the clinic. In the treatment of Gram-negative bacillary sepsis, sulbactam-durlobactam is administered with a 3-h infusion at a schedule of every 4 h. However, this dosing schedule would be prohibitive for administration over many months in patients with MAB lung disease, especially in the outpatient setting. Here, Monte Carlo experiments demonstrated that the inclusion of ceftriaxone shifts the MICs into a range that allows achievement of %T_MIC_ of 50% at dosing schedules of three times a day or less. The sulbactam-durlobactam doses and dose schedules, based on renal function, are shown in Table S6 for inpatients and outpatients. The caveat is that doses selected should be considered merely a first step and will require further clinical validation using real-world evidence.

The mathematical modeling applied in the present study also gave us an insight regarding the duration of therapy. Use of *γ*-modeling allows for pooling data from different experiments, and ranking drugs by how fast they kill MAB. As an example, based on the *γ*-modeling, the SDCEO regimen would need to be administered for 33.75 weeks (7.9 months), to totally eradicate cavitary bacilli (7.0–8.0 log_10_ CFU/lung), to achieve cure rates in >95% of patients (43, 44). Notably, the modeling does not account for re-infection. Since sulbactam-durlobactam demonstrated biologic activity against all four MAB isolates in the HFS-MAB, we consider these findings can be generalized to account for heterogeneity of MAB response in different patients.

We also made a methodological contribution and advancement to Monte Carlo experiments for the two drugs in a fixed-dose combination, including the effects of a third drug (ceftriaxone) on the fixed-dose combination. Sulbactam had a different target exposure from durlobactam, so the question was which drug’s PTA and CFR to use. Renal function had differential effects of sulbactam compared with durlobactam. Here, we introduced methods how to use both drugs’ targets in the combination. We also explored the effect of a third drug, ceftriaxone, which has three effects, (i) on MIC distribution, (ii) on extent of microbial kill and rebound growth, and (iii) on target exposure and dose schedule. These concepts inform us of how we should explore combination therapy of two or more drugs in Monte Carlo experiments in general antibiotic therapeutics.

There are some limitations to our work. The exposure-effect studies were performed with only one HFS-MAB unit per dose due to high cost. Moreover, our results on time-to-extinction were geared towards sterilization of MAB in lung cavities, which is a more difficult test for the experimental regimens (41, 44). In nodular disease, with lower *B_0_*, time to cure could be faster than in cavities (23, 41, 44). Furthermore, the doses we chose using MCE will require further validation in the clinic, using real-world evidence, as was the case with omadacycline and imipenem (21, 45).

To summarize, sulbactam-durlobactam-ceftriaxone trio (“double-double” peptidoglycan biogenesis inhibitors) combination led to faster time-to-extinction compared to sulbactam-durlobactam duo. A further addition of the two protein synthesis inhibitors in the SDCEO regimen suggested that the regimen could potentially shorten the therapy duration. Clinical validation of these findings is warranted.

## MATERIALS AND METHODS

### Bacterial isolates and materials

The reference laboratory isolate (ATCC#19977) was purchased from the American Type Culture Collection. Among the 63 clinical isolates for MIC testing (including two for HFS-MAB experiments), 39 were MAB *subspecies abscessus*, 21 MAB *subsp. massilliense*, and 3 MAB *subsp. bolletii*. Methods for the MIC and time-kill studies are described in detail in Supplemental methods.

### HFS-MAB exposure-effect

The construction and design of the HFS-MAB have been extensively described in the literature (21, 25–29, 38, 45). Here, we combined an exposure-effect study with a dose-scheduling design, using ATCC#19977 reference strain. Drugs were infused over 14 days to mimic the intrapulmonary PKs following human equivalent doses at every X hour (qXh) as follows: 0, 3G q24h, 1.5G q12h, 1G q8h, 4.5G q24h, 1.5G q8h, 6G q24h, 3G q12h, 2G q8h, and 3G q8h. There was one HFS-MAB unit per dose. The drug exposures and sampling details are shown in the Supplemental methods and Table S2.

### HFS-MAB double-β-lactam-β-lactamase inhibitor combination against three MAB clinical isolates

For this set of experiments, we chose ceftriaxone based on the rationale in Supplemental methods and Table S2 (14, 49–51). We examined the effect of sulbactam-durlobactam at target exposures alone, ceftriaxone alone, and sulbactam-durlobactam-ceftriaxone versus non-treated HFS-MAB replicates, using two MAB clinical isolates in the HFS-MAB. In a third HFS-MAB study, we compared the sulbactam-durlobactam-ceftriaxone regimen to combinations of sulbactam-durlobactam-ceftriaxone plus omadacycline and epetraborole (SDCEO), and another regimen in which omadacycline was replaced by minocycline (SDCEM). Epetraborole and omadacycline were chosen because they have the best kill rates below *B_0_* in the HFS-MAB, based on our meta-analysis (24). Sulbactam-durlobactam was administered q12h, while ceftriaxone was delivered q24h. The target concentrations, sampling strategy for PKs, and bacterial burden for all MAB isolates are described in Supplemental methods and Table S4. There were two HFS-MAB units per regimen.

### Mathematical modeling

We modeled CFU/mL using the inhibitory sigmoid E_max_ model for log_10_ CFU/mL versus exposure for microbial kill, while for resistance, we used the quadratic function of Gumbo et al. for each sampling day (3, 52). We used the corrected Akaike Information criteria to identify the sulbactam-durlobactam PK/PD driver (relevant to dosing schedule) for microbial kill and antimicrobial resistance. The data for drug combinations were analyzed based on a system of ordinary differential equations (ODEs) we developed for other mycobacteria in the hollow fiber system and patients, which we previously adapted for MAB (50, 53). This allowed us to calculate the bacteria time to extinction, which is the time to achieve an MAB burden of 10^−2^ CFU/mL, as described in the past (50, 53).

### Monte Carlo experiments (MCEs)

MCE is a tool for *in silico* dose-ranging and dose scheduling experiments to identify the drug dose for use in the clinic. MCEs were performed as described in detail in the Supplemental methods. The population PK parameters used were those of Cammarata et al., which account for between-patient PK variability (54). The probability of target attainment (PTA) is the proportion of patients who will achieve target exposure at each MIC, given the PK variability. The cumulative fraction of response (CFR) to each dose, which is the proportion of all patients in which dose achieves target exposures across the entire MIC distribution, was calculated for each of the four creatinine clearance categories. These were creatinine clearance (i) >90 mL/min, which is normal renal function; (ii) 60–90 mL/min, which is mild renal dysfunction; (iii) ≥30 to <60 mL/min moderate renal dysfunction, and (iv) <30 mL/min, which is severe. We also examined the dose of the co-administered ceftriaxone; the PK model used was that we have published elsewhere for MAC-LD (50). Since durlobactam and sulbactam will have different PK/PD target exposures, we chose the optimal dose as that at which the CFR was ≥90% for both sulbactam and durlobactam (50).

## Data Availability

Upon reasonable request, the raw data for the results presented in the manuscript is available from the corresponding author.

## SUPPLEMENTAL MATERIAL

Supplemental material aac.01437-25-s0001.docx Supplemental methods; Fig. S1 to S4; Tables S1 to S6

ASM does not own the copyrights to Supplemental Material that may be linked to, or accessed through, an article. The authors have granted ASM a non-exclusive, world-wide license to publish the Supplemental Material files. Please contact the corresponding author directly for reuse.
